# Pathobionts in the tumour microbiota predict survival following resection for colorectal cancer

**DOI:** 10.1186/s40168-023-01518-w

**Published:** 2023-05-08

**Authors:** James L. Alexander, Joram M. Posma, Alasdair Scott, Liam Poynter, Sam E. Mason, M. Luisa Doria, Lili Herendi, Lauren Roberts, Julie A. K. McDonald, Simon Cameron, David J. Hughes, Vaclav Liska, Simona Susova, Pavel Soucek, Verena Horneffer-van der Sluis, Maria Gomez-Romero, Matthew R. Lewis, Lesley Hoyles, Andrew Woolston, David Cunningham, Ara Darzi, Marco Gerlinger, Robert Goldin, Zoltan Takats, Julian R. Marchesi, Julian Teare, James Kinross

**Affiliations:** 1 Division of Digestive Diseases, Department of Metabolism, Digestion and Reproduction, Imperial College London, 10th Floor, QEQM Building, St. Mary’s Hospital, Praed Street, London, W2 1NY UK; 2 Department of Gastroenterology, Imperial College Healthcare NHS Trust, London, UK; 3 Section of Bioinformatics, Division of Systems Medicine, Department of Metabolism, Digestion and Reproduction, Imperial College London, London, UK; 4 Department of Surgery & Cancer, Imperial College London, London, UK; 5 Division of Systems Medicine, Department of Metabolism, Digestion and Reproduction, National Phenome Centre, Imperial College London, London, UK; 6 Department of Life Sciences, MRC Centre for Molecular Bacteriology and Infection, Imperial College London, London, UK; 7 Institute of Global Food Security, School of Biosciences, Queen’s University Belfast, Belfast, UK; 8 Cancer Biology and Therapeutics Group, School of Biomolecular and Biomedical Science, UCD Conway Institute, University College Dublin, Dublin, Ireland; 9 Department of Surgery, Faculty Hospital and Faculty of Medicine in Pilsen, Charles University in Prague, Pilsen, Czech Republic; 10 Faculty of Medicine in Pilsen, Biomedical Centre, Charles University in Prague, Pilsen, Czech Republic; 11 Department of Biosciences, Nottingham Trent University, Nottingham, NG11 8NS UK; 12 Translational Oncogenomics Laboratory, The Institute of Cancer Research, 237 Fulham Road, London, SW3 6JB UK; 13 GI Cancer Unit, Department of Medical Oncology, Royal Marsden NHS Foundation Trust, London, UK

**Keywords:** Colorectal cancer, Gut microbiota, Metabolome, Metataxonomics

## Abstract

**Background and aims:**

The gut microbiota is implicated in the pathogenesis of colorectal cancer (CRC). We aimed to map the CRC mucosal microbiota and metabolome and define the influence of the tumoral microbiota on oncological outcomes.

**Methods:**

A multicentre, prospective observational study was conducted of CRC patients undergoing primary surgical resection in the UK (*n* = 74) and Czech Republic (*n* = 61). Analysis was performed using metataxonomics, ultra-performance liquid chromatography-mass spectrometry (UPLC-MS), targeted bacterial qPCR and tumour exome sequencing. Hierarchical clustering accounting for clinical and oncological covariates was performed to identify clusters of bacteria and metabolites linked to CRC. Cox proportional hazards regression was used to ascertain clusters associated with disease-free survival over median follow-up of 50 months.

**Results:**

Thirteen mucosal microbiota clusters were identified, of which five were significantly different between tumour and paired normal mucosa. Cluster 7, containing the pathobionts *Fusobacterium nucleatum* and *Granulicatella adiacens*, was strongly associated with CRC (*P*_FDR_ = 0.0002). Additionally, tumoral dominance of cluster 7 independently predicted favourable disease-free survival (adjusted *p* = 0.031). Cluster 1, containing *Faecalibacterium prausnitzii* and *Ruminococcus gnavus*, was negatively associated with cancer (*P*_FDR_ = 0.0009), and abundance was independently predictive of worse disease-free survival (adjusted *p* = 0.0009). UPLC-MS analysis revealed two major metabolic (Met) clusters. Met 1, composed of medium chain (MCFA), long-chain (LCFA) and very long-chain (VLCFA) fatty acid species, ceramides and lysophospholipids, was negatively associated with CRC (*P*_FDR_ = 2.61 × 10^−11^); Met 2, composed of phosphatidylcholine species, nucleosides and amino acids, was strongly associated with CRC (*P*_FDR_ = 1.30 × 10^−12^), but metabolite clusters were not associated with disease-free survival (*p* = 0.358). An association was identified between Met 1 and DNA mismatch-repair deficiency (*p* = 0.005). *FBXW7* mutations were only found in cancers predominant in microbiota cluster 7.

**Conclusions:**

Networks of pathobionts in the tumour mucosal niche are associated with tumour mutation and metabolic subtypes and predict favourable outcome following CRC resection.

Video Abstract

**Supplementary Information:**

The online version contains supplementary material available at 10.1186/s40168-023-01518-w.

## Introduction

Colorectal cancer (CRC) accounts for 10% of cancers diagnosed worldwide each year [1], and in the UK between 2015 and 2017, there were over 16,000 deaths per year, making CRC the second commonest cause of cancer death [2]. Disease-free survival from CRC is strongly predicted by stage [3]. Other predictors of CRC recurrence after curative resection include lymph node involvement, extra-mural vascular invasion, lymphovascular invasion, perineural invasion and tumour differentiation [4]. However, the evidence base supporting the clinical use of these histological factors is sub-optimal [5], and increasingly molecular markers, such as microsatellite instability (MSI) and mutations in *KRAS* or *BRAF* genes, are used to guide oncological treatment [6–10]. There is an unmet need for precision biomarkers that predict outcome and stratify therapy in CRC patients.

The gut microbiome in CRC serves as a rich target for biomarker discovery, and there is increasing interest in the gut microbiome as a determinant of CRC outcome. Mechanistic studies have implicated a number of mucosal bacteria, including *Fusobacterium nucleatum (F. nucleatum)* [11], specific strains of *Escherichia coli*, [12, 13] *Bacteroides fragilis* [14] and *Peptostreptococcus anaerobius*, [15] collectively referred to as pathobionts, in CRC development and propagation. High abundance of *F. nucleatum* in CRC tissue has been linked to lower levels of T-cell infiltration [16] and worse CRC-specific mortality [17]. However, the cancer mucosal microbiota is highly individualised, dynamic and subject to geographical variation; *F. nucleatum*, for example, is only found at high levels in a small minority of patients with CRC [17], and not all commensal microbiota, e.g. *Bifidobacterium*, appear to have prognostic value [18]. The wide inter-patient variability and enormous redundancy of the gut microbiome argue in favour of a functional understanding of community ecology in CRC [19].

Due to a paucity of longitudinal prospective human studies, there is currently insufficient evidence to draw a direct link between the microbiome and carcinogenesis [19]. Existing cohort studies suffer from two main limitations. Firstly, the retrospective design raises concerns about the effects of unappreciated confounding factors which have been shown to influence the communities in the colonic mucosal and cancer microbiota [20]. Secondly, the focus on a single member of the gut microbial community limits the conclusions that can be drawn on the functional and metabolic contributions of networks of amensalistic and symbiotic microbiota. The driver-passenger model of CRC proposes a dynamic interplay in a genetically susceptible host between evolving communities of microbiota and the developing tumour, orchestrated by a co-metabolite *milieu* in the tumour microenvironment [21] .

In this prospective study, we perform comprehensive metataxonomic, metabolomic and genomic profiling of the CRC mucosa, taking into account covariates known to influence the gut microbiota and CRC outcome. In contrast to previously published work, we show that the presence of pathobiont bacteria in the tumour microbiota is associated with more favourable outcomes following CRC resection, and that these bacteria are associated with discrete metabolic functions and cancer genotypes.

## Methods

### Patient recruitment

A prospective observational study was conducted in patients undergoing CRC resection at two UK cancer centres (Imperial College Healthcare NHS Trust and The Royal Marsden NHS Trust) between November 2014 and January 2017. Ethical approval for this study was provided by the Research Ethics Committee and Health Research Authority (REC reference: 14/EE/0024). Patients’ electronic records were screened for suitability prior to attendance at the hospital.

The inclusion criteria were adult patients (18 years or over) undergoing curative resection for CRC and were able to give informed consent. Exclusion criteria were use of antibiotics within 4 weeks prior to surgery, a personal history of being diagnosed with inflammatory bowel disease or a familial CRC syndrome, previous bariatric surgery and current treatment with enteral or parenteral nutrition.

Patients were recruited prior to undergoing colorectal surgery. Clinical data were collected prospectively, including patient demographics, presenting symptoms, medical and drug history, dietary information, smoking and alcohol intake and neo-adjuvant and adjuvant oncological treatment. Outcomes including disease recurrence and survival were also collected prospectively.

An independently recruited validation sample set was acquired from a cohort of patients recruited between January 2008 and November 2011 at the University Hospital and Faculty of Medicine in Pilsen, Charles University, Czech Republic, under ethical and regulatory approval from the Ministry of Health in the Czech Republic (approval number: 10230–3).

### Tissue sampling and processing

The technique for sampling of tissue was equivalent in both cohorts, and the size of specimens used for analysis was similar. Immediately after resection, the fresh surgical specimen was opened by a histopathologist. Sterile water was washed gently over the mucosal surface to remove adherent faecal matter. Tissue samples were cut from the tumour and normal mucosa at least 10 cm from the tumour site using a sterile blade. Tissue samples were divided into sub-aliquots between 50 and 100 mg, and the individual aliquots were stored in cryovials at − 80 °C.

### DNA extraction from tissue samples

Total genomic DNA was extracted from the samples using the PowerLyzer PowerSoil DNA extraction kit (Qiagen, Hilden, Germany; previously by MOBIO) following manufacturer’s instructions with the following modifications. A single tissue sub-aliquot for each sample was used for DNA extraction. Briefly, beads were added to each sample tube with a bead beating buffer solution and sodium dodecyl sulphate solution and gently vortexed. The samples were placed in a Bullet Blender Storm bead beater for 3 min at power setting 8 to cause homogenisation and cell lysis. The tubes were centrifuged at 10,000 × *g* for 3 min at room temperature, and 500 µl of supernatant was obtained. Subsequent steps were according to the manufacturer’s protocol. The resulting DNA solution was divided into 20-µl aliquots of extracted DNA suspended in TE buffer, which were stored at − 80 °C pending downstream analysis. Total DNA yield per sample was measured using the Qubit 2.0 Fluorometer (Life Technologies).

### Metataxonomic analysis of tissue microbiota

16S rRNA gene sequencing was performed at Research and Testing Laboratory, Texas, USA. Samples were amplified for sequencing in a two-step process, using a primer set previously described [22]. The forward primer was constructed with (5′-3′) the Illumina i5 sequencing primer (TCGTCGGCAGCGTCAGATGTGTATAAGAGACAG) and the gene-specific primer combination (28F-YM: GAGTTTGATYMTGGCTCAG + 28F-Borrelia: GAGTTTGATCCTGGCTTAG + 28F-Chloroflex: GAATTTGATCTTGGTTCAG + 28F-Bifido: GGGTTCGATTCTGGCTCAG) in a 4:1:1:1 ratio. The reverse primer was constructed with (5′-3′) the Illumina i7 sequencing primer (GTCTCGTGGGCTCGGAGATGTGTATAAGAGACAG) and the gene-specific reverse primer (388R: TGCTGCCTCCCGTAGGAGT) [22]. Amplifications were performed in 25-µl reactions with Qiagen HotStar*Taq* Master Mix (Qiagen Inc., Valencia, CA, USA), 1 µl of each 5-µM primer and 1 µl of template. Reactions were performed on ABI Veriti thermocyclers (Applied Biosytems, Carlsbad, CA, USA) under the following thermal profile: 95 °C for 5 min, 35 cycles of 94 °C for 30 s, 54 °C for 40 s, 72 °C for 1 min, followed by one cycle of 72 °C for 10 min and 4 °C hold. Products from the first stage amplification were added to a second PCR based on qualitatively determined concentrations. The second PCR was performed using the Illumina Nextera XT Index Kits. Primers for the second PCR were designed based on the Illumina Nextera PCR primers as follows: forward-AATGATACGGCGACCACCGAGATCTACAC[i5index]TCGTCGGCAGCGTC and reverse-CAAGCAGAAGACGGCATACGAGAT[i7index]GTCTCGTGGGCTCGG. The second stage amplification was run under the following thermal profile: 95 °C for 5 min, 10 cycles of 94 °C for 30 s, 54 °C for 40 s, 72 °C for 1 min, followed by one cycle of 72 °C for 10 min and 4 °C hold.

Amplification products were visualised with eGels (Life Technologies, Grand Island, NY, USA). Products were pooled in equimolar concentrations, and each pool was size selected in two rounds using Agencourt AMPure XP (Beckman Coulter, Indianapolis, IN, USA) in a 0.75 ratio for both rounds. Size-selected pools were quantified using the Qubit 2.0 Fluorometer (Life Technologies) and loaded on an Illumina MiSeq (Illumina, Inc. San Diego, CA, USA) 2 × 300 flow cell at 10 pM. Fastq files were generated for the forward and reverse reads for each sample.

### Analysis of 16S rRNA gene amplicon sequencing data

Data analysis was initially performed in mothur v.1.39.5 [23, 24] (http://www.mothur.org/wiki/MiSeq_SOP#OTU-based_analysis). The forward and reverse reads were paired using make.contigs. Ambiguous bases, excessively long homopolymers and those sequences that were longer than 365 base pairs (97.5% tile) or shorter than 335 base pairs (2.5% tile) were removed. Duplicate sequences were removed using unique.seqs. The sequences were aligned to a customised reference (SILVA www.arb-silva.de/), and poorly aligned sequences were removed. Filter.seqs was used to remove empty columns from alignments, and pre.cluster was implemented to remove chimaeras and sequences due to pyrosequencing errors. Split.abund command was used to split the sequences into rare and abundant groups, and the sequences were assigned to taxonomy against the 16S rRNA gene reference of RDP v10. Average neighbour clustering was used. Species other than bacteria (mitochondria, archaea, etc.) were removed using remove.lineage. The sub.sample command was used to normalise reads to 7500, ensuring identical sequencing depth per sample. This resulted in the elimination of 24 samples (7%) from the UK cohort and 15 samples (10%) from the Czech cohort with read counts less than 7500. A minimum coverage threshold of 99.5% was set, which was satisfied by all remaining samples. Data for total number of reads per sample, before and after QC filtering, and coverage are presented in the supplementary information (Table S1). Alpha (Shannon and Chao1), and beta diversity (weighted UniFrac) indices were calculated in mothur. Sequences were assigned to taxonomy against the 16S rRNA gene reference of RDP v10. As a secondary assignment method, where possible, species assignment was performed using NCBI BLAST for microbial genomes [25], with a minimum sequence similarity threshold of 97%. Target bacteria culture and bacterium-specific qPCR were subsequently performed to confirm the identity of key bacteria (see supplementary information).

### Metabolomics of tissue extracts

Aqueous and organic phase tissue extractions were performed for hydrophilic interaction liquid chromatography (HILIC) and reversed-phase chromatography (RPC) ultra-performance liquid chromatography-mass spectrometry (UPLC-MS) analyses respectively. Full details of the methods for aqueous and organic phase extractions can be found in the supplementary information. The protocol herein was adapted from those previously published [26, 27].

### UPLC-MS analysis

Lipid profiling of the organic phase tissue extract and HILIC-LC–MS of the aqueous phase tissue extract were performed using the same experimental UPLC-MS conditions as described previously [28]. For HILIC-based chromatographic retention and separation of polar molecules, a 2.1 × 150 mm Acquity BEH HILIC column (Waters Corp., Milford, MA, USA) was used at 40 °C operational temperature. The solvent system was acetonitrile with 0.1% v/v formic acid and 20-mM ammonium formate in water with 0.1% v/v formic acid. A flow rate of 0.6 ml/min was used for sample loading and gradient elution. Sample handling was performed with a Waters 2777C sample manager (Waters Corp., Milford, MA, USA). Chromatography was done on an ACQUITY UPLC (Waters Corp., Milford, MA, USA) which was coupled via a Zspray electrospray ionisation (ESI) source to a high-resolution orthogonal acceleration time‐of‐flight mass spectrometry Xevo G2-S oaTOF MS (Waters Corp., Manchester, UK) and operated in positive and negative ion modes.

#### Quality control (QC) samples

For quality control and quality assurance, two types of QC samples (long-term reference, LTR, and study reference, SR) were injected at regular intervals throughout the run and used to support the analytical quality assessment of the data as described previously [28]. In addition, a mixture of authentic reference materials acting as internal standards (IS) and method reference (MR) were added to SR and LTR samples to monitor data quality during acquisition.

### LC–MS data extraction

Vendor MS data files in the Waters.RAW data files were converted to the open mzML [29] format using Proteowizard’s *msconvert* [30]. A denoising step was applied during conversion, which consists of removal of all centroid signals with intensity below 100 counts. Untargeted peak detection, alignment, grouping, integration and deisotoping were performed using Progenesis QI 2.1 software (Waters Corp., Manchester, UK). Run order and batch correction of intensity drifts and feature filtering were performed using the nPYc-Toolbox [31, 32]. The SR dilution series and repeated SR injections were used to filter features with a Pearson correlation with dilution below 0.7 and with a relative standard deviation on the SR injections above 30%, as described in Lewis et al. [33].

Chemical identity was assigned by matching accurate mass and tandem mass spectrometry (MS/MS) fragmentation (of the protonated molecule) measurements to reference spectra using an in‐house database constructed from analysis of authentic reference materials. Where authentic reference materials were commercially available, they were used to generate definitive molecular identification by direct matching of chromatographic and spectral qualities (including accurate mass, MS/MS spectra and isotopic distribution) to those observed in the profiling data and subsequent targeted MS/MS experiments. Targeted extraction and integration of pre-annotated features were performed using the R package peakPantheR [34] .

### Cancer hotspot panel

Genes of interest in CRC (*PIK3CA*, *APC*, *HRAS*, *TP53*, *BRAF* and *KRAS*) were sequenced in 30 tumour samples at the NHS molecular pathology laboratory, Hammersmith Hospital, London UK, using the Ion AmpliSeq Cancer Hotspot Panel v2 (ThermoFisher) according to manufacturer specifications (https://www.thermofisher.com/document-connect/document-connect.html?url=https%3A%2F%2Fassets.thermofisher.com%2FTFS-Assets%2FLSG%2Fmanuals%2FMAN0006735_AmpliSeq_DNA_RNA_LibPrep_UG.pdf&title=VXNlciBHdWlkZTogSW9uIEFtcGxpU2VxIExpYnJhcnkgS2l0IDIuMA = =).

### Tumour exome sequencing

Full exome sequencing was performed on tumour samples (mean depth of 142–162 ×) and paired normal mucosa samples (mean depth 87–105 ×) from nine patients. An Agilent human exome sequencing kit was used. Full methods are described in the supplementary methods.

### Data integration and statistical analysis

Microbiota alpha diversity was analysed in GraphPad Prism v8 using Wilcoxon matched-pairs signed-rank tests. Beta diversity was analysed using PERMANOVA (R-vegan function adonis). Individual operational taxonomic units (OTUs) were combined at the species level if names were identical, unclassified OTUs (not allocated to specific species) were combined at the genus level and any unclassified bacteria were removed. Last, any OTUs with less than 25% of non-zero values were removed; the final data contains 94.05% of the total variance of the raw data. Following these curation steps, a median of 97.4% of reads remained per sample (*IQR*: 93.2–99.1). Fatty acid (FA) metabolites and metabolites with FA chains from the targeted metabolomics data were also combined based on FA classes as short-chain FA (2–6 carbons), medium-chain FA (7–12 carbons), long-chain FA (13–21 carbons) and very-long-chain FA (22 + carbons).

Partial correlations, using Spearman-rank-based correlation, were calculated for the 16S rRNA gene and metabolomics data individually (tumour samples only); these were corrected for potential confounding factors (population factors: age, sex, ethnicity; lifestyle factors: body mass index, smoking (current), smoking (ever), alcohol, red meat eater; clinical: Charlson score, protein-pump-inhibitor use, tumour location, neoadjuvant treatment, mucinous tumour type, tumour differentiation, extra-mural vascular invasion (EMVI) status, American Joint Committee on Cancer (AJCC) stage). Significance of each partial correlation was determined based on a permutation strategy where the data of each variable was scrambled independently from other variables, and partial correlations were recalculated. This process was repeated 1000 times, and a partial correlation of two variables was considered significant if less than 5% of the random permutations had higher (in the case of *r* > 0) or lower (for *r* < 0) correlations. The partial correlations reported are those that were significant after the permutation adjustment to control for multiple testing.

The clustering was obtained using hierarchical cluster analysis (correlation distance, average linkage), and the optimal number of clusters was determined by calculating the modularity of the splitting and comparing this with 1000 permutations of the correlation data [35]. The optimal modularity is the splitting where the modularity is a local maximum and the most significantly different from the random permutations (adjusted for multiple testing using the Benjamini–Hochberg FDR). If this does not yield a single optimum, the splitting is chosen as the one that is most different from the random alternatives (and *FDR* < 5%).

Throughout the analysis, the included variables and obtained clustering are given for the UK cohort and applied to the validation (Czech) cohort. The modularity (and 1000 random permutations) is recalculated for the validation cohort and significance assessed at the splitting level obtained from the training (UK) data.

The variables within each cluster were combined by calculating the first left singular vector (first principal component score) of the data of those variables. These latent representations of each cluster were used to test the difference between tumour samples and (paired) normal tissue (paired *t*-test). 16S rRNA gene clusters were correlated with the metabolites (controlled for the sample confounders), and significance was determined based on the same permutation strategy as used for the initial analysis. All calculations were performed in MATLAB v8.3 (the MathWorks, Natick, USA). The codes are available from GitHub (https://github.com/jmp111/CRC) and the processed data from Zenodo (https://doi.org/10.5281/zenodo.7326674).

To test associations between microbiota and metabolite clusters and target gene mutations, Mann–Whitney tests were performed.

A partial least squares discriminant analysis model was calculated using Monte Carlo cross-validation with 1000 iterations [36] for the metabolomics data accounting for the repeated measures design (tumour vs paired normal tissue), to avoid samples from the same individual being split between training and test sets. Moreover, these data are centred for each individual to allow the model to focus on within-person differences that reflect the differences between the tumour and paired normal tissue. The targeted data (training sets) were auto-scaled (mean centering followed by unit-variance scaling) in each iteration, and scaling factors (mean, standard deviation) were applied to each independent test set. Results are represented as scores from the model for samples and as a skyline plot (− log10(FDR) × sign(β)) for variables. A *p*-value is calculated for each variable across the 1000 models (mean) using 25 bootstrap models each to evaluate the regression coefficient (*β*) variance (25,000 models). These were then adjusted using the FDR.

Finally, we conducted survival analysis with disease-free survival used as the outcome of interest. Tumour samples for each patient were assigned to ‘high’ or ‘low’ for microbiota clusters 1 and 7 and metabolome clusters 1 and 2 by dichotamising at the median for the relative abundance of the summed data from each cluster. Kaplan–Meier curves and differences in survival were calculated with the log-rank test. Cox proportional hazard models were used to investigate the associations, controlling for confounders including age, sex, body mass index (BMI), tumour anatomical location (right colon, left colon and rectum), AJCC stage, extra-mural vascular invasion and chemotherapy. Analysis was performed using the ‘survival’ (v3.2.10) and ‘survminer’ (v0.4.9) packages in R. All statistical tests were two-sided, and statistical significance was determined as a *p*-value < 0.05.

## Results

### Patient demographics and histological findings

Seventy-four CRC patients were included in the analysis of the UK cohort. Demographic, clinical and histopathological characteristics are shown in Table 1. Demographic and histological information for 61 CRC patients in the Czech validation cohort is presented in Table S2.

**Table 1:** Demographic, clinical and histological data of UK cohort

N	74
Male:female	41:33
Median BMI (range)	26.5 (15.1–36.0)
Median age (range)	70 (36–91)
Ethnicity	
*White*	56
*Asian/Asian British*	11
*Black/Black British*	6
*Other*	1
Smoking	
Never smoker	33
Current smoking	9
Previous smoking	32
Alcohol	
*Non-drinker*	29
*1–10 units per week*	25
*10–20 units per week*	13
*> 20 units per week*	7
Vegetarian	3
Red meat	
*< 2 portions/week*	40
*2 or more portions/week*	34
Family history of CRC	16
Charlson score (median)	5
Proton-pump Inhibitor use	17
Tumour site	
*Rectum*	23
*Sigmoid & recto-sigmoid*	15
*Descending*	2
*Splenic flexure*	3
*Transverse*	8
*Hepatic flexure*	2
*Caecum & ascending colon*	21
Neo-adjuvant treatment	
*None*	68
*Long-course chemoradiotherapy*	5
*Chemotherapy alone*	1
T stage	
*T1/2*	18
*T3*	42
*T4*	14
N stage	
*N0*	51
*N1*	18
*N2*	5
M stage	
*M0*	71
*M1*	3
AJCC stage	
*I*	14
*II*	37
*III*	20
*IV*	3
Differentiation	
*Well*	3
*Moderate*	54
*Moderate–poor*	4
*Poor*	13
DNA mismatch repair deficiency$	14/46
Extra-mural venous invasion	27
Lymphovascular invasion	28
Tumour budding	47

$MMR classification available on 46 of 74 tumour samples

### Microbiota clustering identifies distinct bacterial communities linked to CRC outcome

**Fig. 1:**
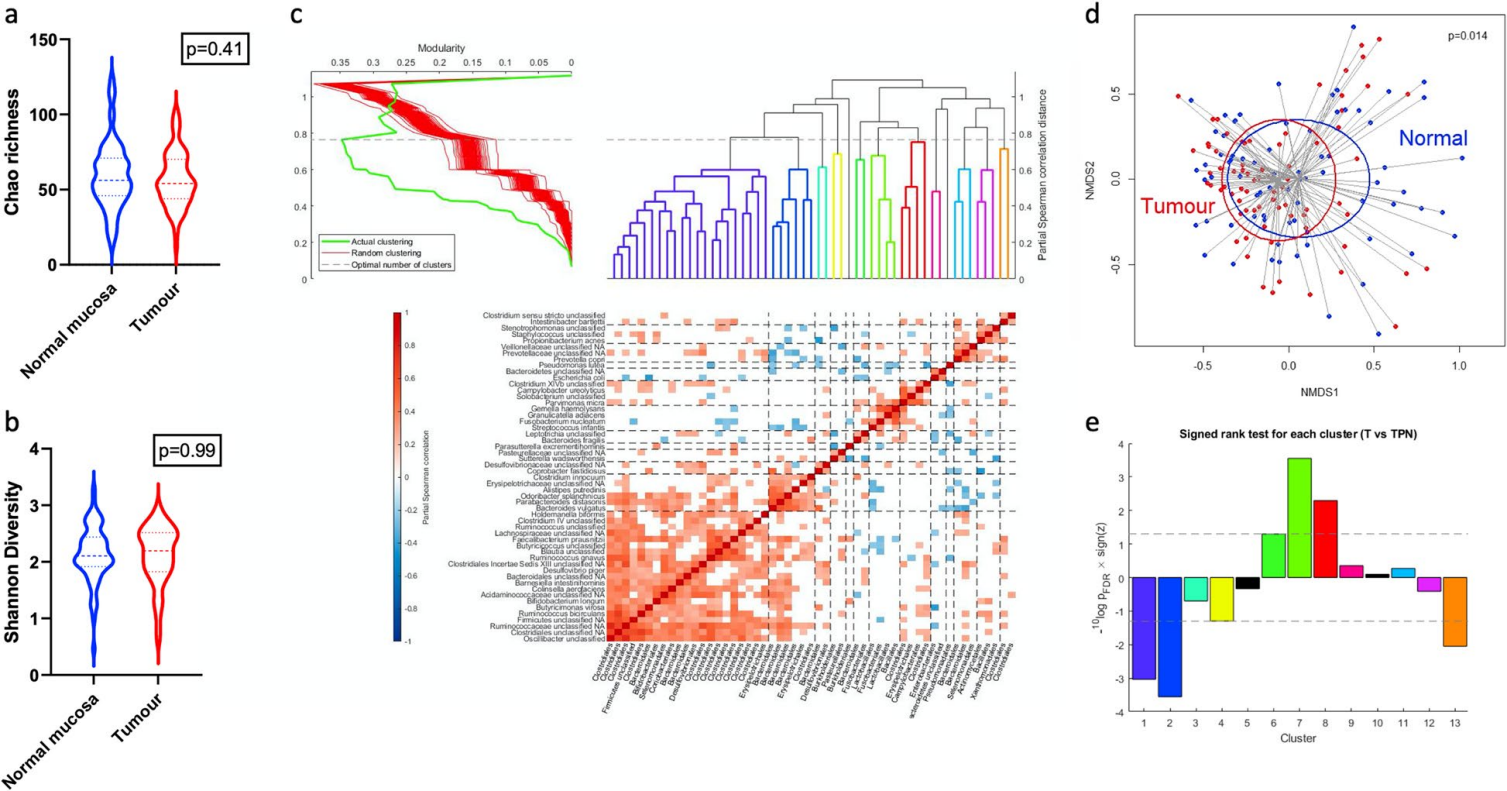
Microbiota analysis of the colorectal cancer mucosa **a** Chao richness paired comparison between tumour and paired normal mucosa (Wilcoxon matched-pairs signed-rank test *p* = 0.41). **b** Shannon diversity paired comparison between tumour and paired normal mucosa (Wilcoxon matched-pairs signed-rank test *p* = 0.99). **c** Hierarchical clustering of microbiota. *Y*-axis labels are species or higher taxonomic rank if species data is not known; *x*-axis labels show the order. **d** Beta diversity displayed as a nonmetric dimensional scaling (NMDS) plot of weighted UniFrac distances for normal mucosa (blue) and tumour (red). Ellipses drawn to indicate 95% confidence intervals. *R*^2^ = 0.027; *p* = 0.014 (adonis PERMANOVA). **e** Paired comparison between tumour and tumour-paired normal samples for each identified microbiota cluster

To determine differences in the mucosal microbiota associated with CRC primary tumours, we undertook a paired analysis of diversity metrics comparing tumour samples with normal adjacent mucosa. There was no significant difference in Chao richness or Shannon diversity between tumour and paired normal mucosa (Fig. 1 a and b; *p* = 0.41 & 0.99, respectively). Weighted UniFrac distances were used to perform nonmetric multidimensional scaling (NMDS) of samples (Fig. 1d). Applying a PERMANOVA test demonstrated significant difference in beta diversity between tumour and paired normal mucosa (*R*^2^ = 0.027; *p* = 0.014).

Clustering analysis was performed on tumour samples to determine the optimal splitting of bacterial clusters. This analysis defined thirteen bacterial clusters (Fig. 1c). The largest cluster was cluster 1 (21 OTUs). The most abundant OTUs in cluster 1 were *Ruminococcus gnavus* (proportion of cluster = 0.39), *Faecalibacterium prausnitzii* (0.23) and *Blautia* species (0.05). Other notable clusters were cluster 2 (six OTUs) including *Bacteroides vulgatus* (proportion of cluster = 0.80) and *Parabacteroides distasonis* (0.13) and cluster 7 (four OTUs) including *Fusobacterium nucleatum* (0.48), *Gemella haemolysans* (0.07) and *Granulicatella adiacens* (0.05). Comparison of tumour samples against their paired normal mucosal samples revealed five of the thirteen clusters to be significantly different (Fig. 1e). Cluster 7 and cluster 8 were strongly associated with tumour (*P*_FDR_ = 2.80 × 10^−4^ & *P*_FDR_ = 5.07 × 10^−3^, respectively), and clusters 1 and 2 had strong negative associations with tumour (*P*_FDR_ = 9.25 × 10^−4^ and *P*_FDR_ = 2.80 × 10^−4^, respectively). Cluster 13 (containing *Intestinibacter bartlettii* and unclassified *Clostridium *sensu stricto) was also negatively associated with cancer (*P*_FDR_ = 0.008). To validate the UK clustering results, an identical approach was applied to the 61 Czech CRC samples (Fig. S1). Again, thirteen clusters were identified using this algorithm. Bacteria-specific qPCR confirmed the identity at species level of key bacteria in cluster 1: *Ruminococcus gnavus* and *Faecalibacterium prausnitzii* and in cluster 7: *Fusobacterium nucleatum* and *Granulicatella adiacens* (Fig. S2).

Harnessing the statistically significant clusters of microbiota identified herein, we tested the hypothesis that the mucosa-associated bacterial ecological niche of CRCs would be predictive of disease outcome following primary resection. A total of 127 patients (all UK based) were included in the analysis of microbiota clusters and outcome. This cohort included the 74 patients from the aforementioned UK cohort, and an additional 53 patients in whom tumour microbiota data, but not tumour metabolome data, were available. Characteristics of the 127 patients analysed are shown in Table 2. Five patients had stage 4 disease. Two of the five had peritoneal metastatic disease which was excised at primary surgery. Another two had peritoneal metastasis that was not resected, and the patients received adjuvant chemotherapy. One patient had lung metastases which were not amenable to metastasectomy. In total, forty patients (31.5%) received adjuvant chemotherapy. Anatomical distribution of cancers, AJCC stage, tumour differentiation and EMVI status were represented in proportional numbers to the 74-patient UK cohort. The median period of follow-up was 50 months (interquartile range 34–60). Over the course of follow-up, 90 patients (70.9%) remained alive and had no recurrence of CRC, and 37 patients (29.1%) suffered recurrence of CRC and/or died.

**Table 2:** Characteristics of 127 CRC patients in Cox proportional hazards outcome analysis

Median follow-up in months (IQR)	50 (34–60)
CRC recurrence or death	37 (29.1%)
Median age at CRC resection	70 (36–91)
Male:female	68:59
Tumour location	
*Right colon*	52
*Left colon*	23
*Rectum/recto-sigmoid*	52
Adjuvant chemotherapy	40 (31.5%)
AJCC stage	
*I*	23
*II*	56
*III*	43
*IV*	5
Differentiation	
*Well*	4
*Moderate*	96
*Moderate–poor*	5
*Poor*	22
EMVI	47 (37.0%)

**Fig. 2:**
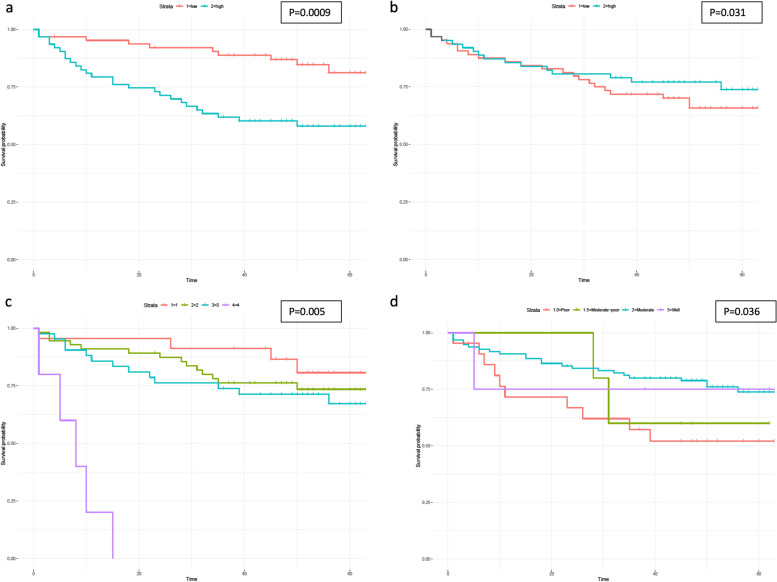
Survival analysis demonstrates prognostic utility of colorectal cancer mucosal microbiota clustering. Kaplan–Meier curves illustrating the difference in disease-free survival in groups stratified by CRC mucosal abundance of microbiota (**a** cluster 1 microbiota; **b** cluster 7 microbiota) and by established prognostic factors (**c** AJCC stage; **d** tumour differentiation). Time is measured in months since primary tumour resection. For microbiota clusters, individuals were split at the proportional median and classified as “low” (red) and “high” (blue) expressors of each cluster of microbiota. Log-rank test used to generate *p*-values

In univariate Cox regression analysis, treating cluster proportional abundance as a continuous variable, lower abundance of cluster 1 and higher abundance of cluster 7 microbiota in tumour samples were significantly associated with better disease-free survival (*p* < 0.0001 and *p* = 0.040, respectively). The associations between microbiota clusters 1 and 7 with disease-free survival were tested in multivariable analysis. Confounding variables were first subjected to separate univariate Cox regression, revealing that AJCC stage (treated as a categorical variable: stages 1–4) and tumour differentiation (categorical variable: poor, moderate-poor, moderate and well differentiated) were associated with outcome (*p* = 0.005 & *p* = 0.036, respectively). Other variables including age, sex, BMI, use of adjuvant therapy, anatomical location of primary tumour and EMVI status were not significantly associated with outcome (Table S3). In multivariable analysis, accounting for the significant covariates, microbiota cluster 1 (hazard ratio (HR) 1.26; 95% *CI* 1.10–1.45; = 0.0009) and cluster 7 (*HR* 0.60; 95% *CI* 0.38–0.96; *p* = 0.031) remained significantly associated with outcome (Fig. 2 a and b).

### Metabolomic and tumour genomic analyses deliver insights into relationship between the microbiota and CRC outcome

**Fig. 3:**
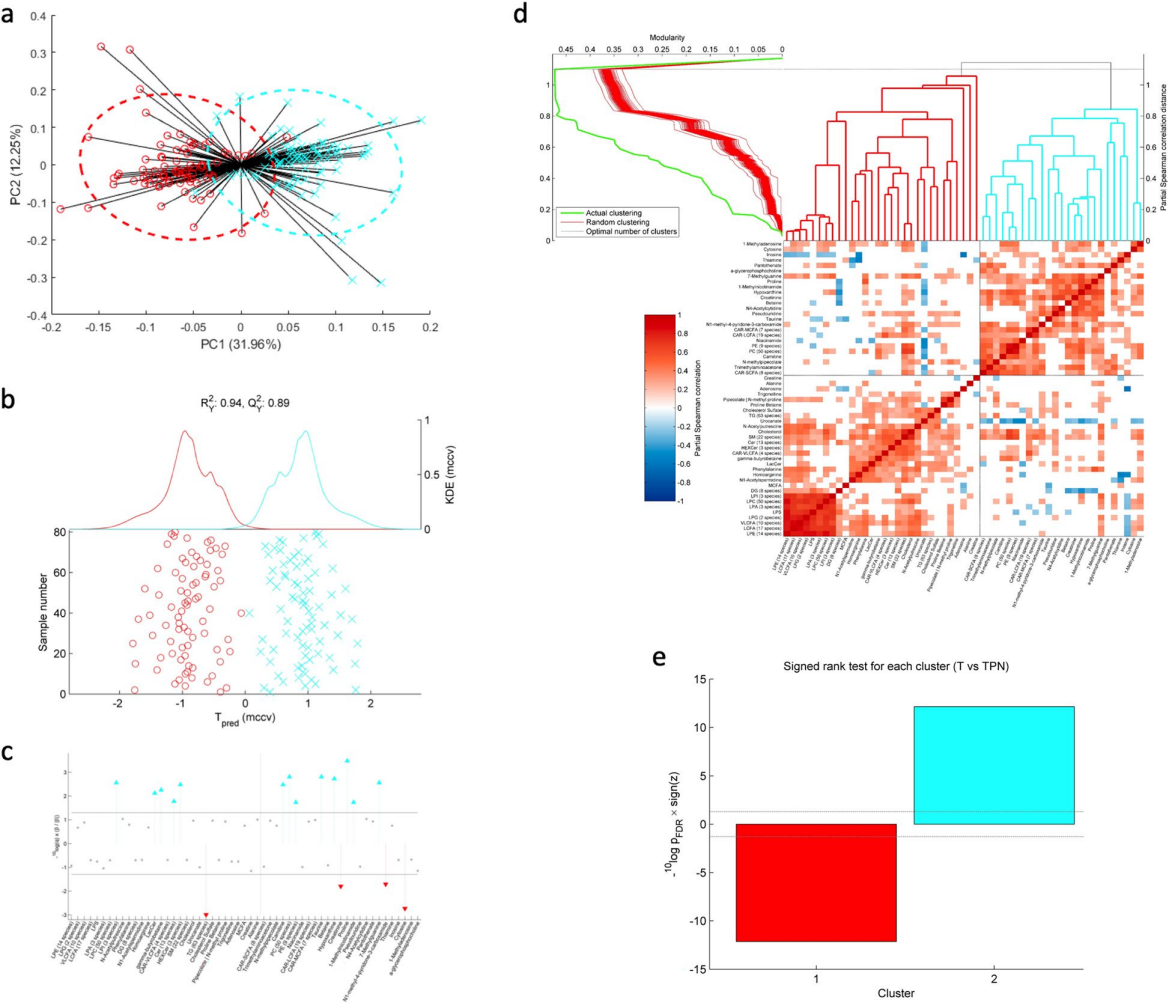
Metabolomic analysis of the colorectal cancer mucosa. **a** Principal components analysis of metabolomic data for tumour (blue) and paired normal tissue (red). **b** Cross-validated scores plot of the repeated measures partial least squares discriminant analysis model (goodness-of-fit *R*^2^*Y* = 0.95, goodness of prediction *Q*^2^*Y* = 0.89). Tumour represented in blue and paired normal tissue in red. **c** Skyline plot indicating metabolites which are significantly higher in tumour (upward blue arrows) or higher in paired normal mucosa (downward red arrows). The dotted horizontal lines indicate the cut-off for the *P*_FDR_ at 5%. **d** Hierarchical clustering of metabolites. Fatty acids are grouped in SCFA, MCFA, LCFA and VLCFAs. **e** Paired comparison between tumour and paired normal mucosa samples for each identified metabolite cluster

To gain a deeper understanding of the factors driving our observed association between the tumoral microbiota and disease-free survival, we performed multi-omic analysis of the CRC mucosal microbiota. First, multivariate principal components analysis (PCA) and partial least squares discriminant analysis (PLS-DA) were undertaken comparing metabolomic data from tumour (T) samples and paired normal (TPN) samples from the 74 UK CRC patients (Fig. 3 a and b). In unsupervised PCA, separation between T and TPN samples is evident in the first principal component (Fig. 3a), and PLS-DA modelling (Fig. 3b) performed highly robustly in separating T and TPN (*R*^2^*Y* = 0.95; *Q*^2^*Y* = 0.89). A skyline plot was derived to demonstrate metabolites which were significantly distinct between T and TPN (Fig. 3c). Tumour-associated metabolites included phospholipids (phosphatidylcholines (PC), phosphatidylethanolamines (PE) and lysophosphatidylinositol (LPI)), sphingolipids (ceramides, hexosylceramides and lactosylceramides), amino acids (alanine, taurine and proline) and purine derivatives (hypoxanthine and 7-methylguanine). Metabolites enriched in paired normal mucosa included triglycerides, creatinine, *N*1-methyl-4-pyridone-3-carboxamide and cytosine.

Using the same bioinformatic approach as was used to cluster the microbiota, clustering analysis was performed on the tumour (T) metabolite data, which defined the optimal number of clusters as two (Fig. 3d). Notable components of cluster Met 1 included FAs, medium-chain (MCFA), long-chain (LCFA) and very-long-chain (VLCFA) species, ceramides and lysophospholipids. Cluster Met 2 included a large number of PC species, nucleosides, amino acids and carnitines. Paired comparison of the two metabolite clusters between tumour and paired normal mucosa revealed a highly significant paradoxical association (Fig. 3e). Metabolite cluster 1 was strongly negatively associated with tumour (*p* = 2.61 × 10^−11^), and metabolite cluster 2 was strongly positively associated with tumour (*p* = 1.30 × 10^−12^). The dichotomous clustering into two groups of metabolites seen in the UK data was mirrored in the Czech data (Fig. S5). Unlike the microbiota, in Cox proportional hazard analysis, tumoral metabolite cluster abundance was not associated with disease-free survival (*p* = 0.358).

**Fig. 4:**
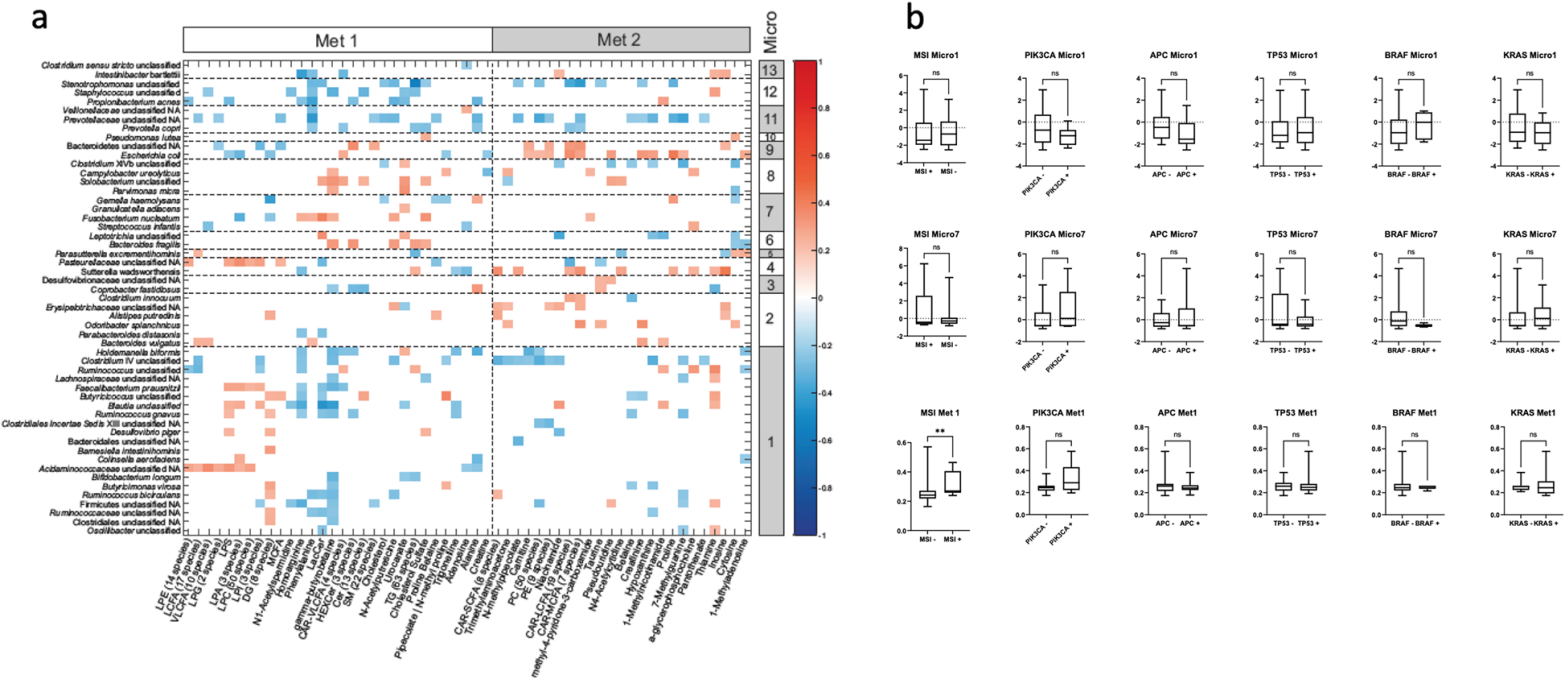
Integration of the colorectal cancer mucosal microbiota, metabolome and tumour driver mutations. **a** Correlation between individual microbiota and metabolites. Positive correlations shown in shades of red; negative correlations in shades of blue. Only statistically significant correlations are shown. Microbiota and metabolites are ordered by the clustering from the individual dataset-specific analyses. Microbiota clusters are labelled along the right side of the figure and metabolite clusters along the top with dotted lines indicating division of clusters. **b** Box and whisker plots showing median and 95% confidence intervals for cluster proportions in patients with ( +) and without ( −) target mutations of interest. **p-value < 0.01

Next, an integration of paired (metataxonomic-based) microbiota and metabolome data was performed to identify bacteria–metabolite associations in the CRC mucosa (Fig. 4a). Additionally, a network analysis linking bacteria with genes encoding enzymatic functions involving the identified metabolites was derived from searching the KEGG database (Fig. S4). Several notable associations were found. Gamma-butyrobetaine was correlated with cluster 7 microbiota that includes *Fusobacterium nucleatum* and negatively correlated with cluster 1 microbiota including *Ruminococcus gnavus*, *Blautia*, *Faecalibacterium prausnitzii* and *Bifidobacterium longum*. Gamma-butyrobetaine is the metabolic precursor of carnitine biosynthesis and is also known as a potential source of carbon and nitrogen for bacteria [37]. The related compound 3-methyl-4-(trimethylammonio)butanoate is bacterially derived from anaerobic commensals in the gut and thought to be metabolically active in the central nervous system through inhibition of FA oxidation [38]. Lactosylceramide (LacCer) was positively correlated with *Fusobacterium nucleatum* and unclassified *Leptotrichia* and negatively correlated with *Ruminococcus gnavus*, *Blautia* spp., *Faecalibacterium prausnitzii* and *Parabacteroides distasonis*. Intestinal epithelial cells express LacCer which binds both commensal and pathogenic bacteria [39, 40]. In a murine context, the accumulation of LacCer has been implicated in the development of colitis-associated CRCs [41]. Lysophosphatidic acid (LPA) was positively correlated with *Collinsella aerofaciens*, *Blautia* spp. and *Faecalibacterium prausnitzii*. Aberrant LPA production and signalling have been linked to neoplasia and cancer progression [42]. Phenylalanine was positively correlated with *Fusobacterium nucleatum* and *Bacteroides vulgatus*. There is evidence that phenylalanine is required for the growth of anaerobes including *F. nucleatum* and *Porphyromonas gingivalis* [43].

Associations were tested between the microbiota and metabolome clusters with DNA mismatch repair (MMR) status (analysed routinely by immunohistochemistry during histopathological analysis) and tumour driver mutations from tumour hotspot analysis (Fig. 4b). A positive association was found between Met cluster 1 and MMR-deficient tumours (*p* = 0.005). No associations were found with other mutations including *APC*, *PIK3CA*, *KRAS* and *TP53*, and we did not identify mutations found exclusively to be associated with individual microbiota. Recognising that MMR deficiency is an important determinant of CRC outcome, we further validated our finding of an association between microbiota cluster 7 and disease-free survival in 79 patients with recorded MMR status (*n* = 17 MMR-deficient tumours). Accounting for all covariates in the prior Cox proportional hazard’s analysis and including MMR status, microbiota cluster 7 remained associated with disease-free survival (*p* = 0.042).

Finally, in an exploratory analysis of the tumours which were analysed by whole exome sequencing (demographics Table S4), *FBXW7* gene mutations (*n* = 3) were exclusively found in tumoural samples in which microbiota cluster 7 was dominant (Fig. S5). Tumour samples with high mutation burden and with mutations in mismatch repair genes were dominated by various microbiota and metabolome clusters, although *MSH6* gene mutations were present only in tumour samples in which metabolome cluster 1 was dominant.

## Discussion

This multi-omic prospective study is the first comprehensive combined analysis of the CRC mucosal microbiota and tumour metabolome. In patients undergoing surgical resection, we show that the bacterial community composition of the CRC mucosal microbiota, but not the CRC metabolome, is predictive of disease-free survival, independent of variables including AJCC stage, tumour location, adjuvant oncological treatment and tumour MSI status. Our significant finding is that higher tumour abundance of a cluster of microbiota (cluster 7), including pathobiont genera *Fusobacterium*, *Granulicatella* and *Gemella*, is independently associated with better outcomes following primary resection questions orthodox thinking on the involvement of the gut microbiota in CRC prognosis. Previous studies have associated high *Fusobacterium nucleatum* abundance, measured individually, with unfavourable outcomes [17]. Interestingly, however, a small retrospective study with short follow-up identified a non-significant association between a bacterial co-abundance group containing *Fusobacterium* and longer survival [44]. A plausible interpretation of our results is that the higher abundance of cluster 7 pathobiont microbiota may precipitate a more active immune response to CRC. Thus, in the aftermath of primary CRC resection, immune memory against such cancers may persist and remain vigilant against local or distant recurrence. In contrast, tumours abundant in cluster 1 microbiota might fail to induce immune memory, and thus, recurrence in such cases goes un-checked by host immunity. It has been shown that higher density of intratumoral infiltrates of CD8^+^ cytotoxic T lymphocytes is associated with reduced CRC recurrence and better prognosis, independent of cancer stage [45]. The subsequent validation of the Immunoscore [46] as a reliable estimate of risk of CRC recurrence raises the question of whether exogenous factors, such as the gut microbiota, might be contributing to instigation of post-resection immune surveillance. Existing data suggest that *F.* *nucleatum* is inversely associated with intratumoural CD3^+^ T-cell density, but not associated with density of CD8^+^ T cells [16], although the contribution of the wider gut microbiota ecosystem has yet to be explored. For example, *Bacteroides fragilis*, another pathobiont, can trigger an inflammatory pro-carcinogenic cascade via its eponymous toxin [47].

A recent large study of the faecal metabolome demonstrated the potential utility of metabolites as biomarkers in diagnosing CRC [48], but concomitant studies on the CRC mucosal metabolome, and its relevance to prognosis, are lacking; existing knowledge is restricted to studies derived from small and/or retrospective patient cohorts [49–51]. The analysis of the tumour mucosal metabolome in the current study has revealed 14 classes (a total of 85 metabolites) of CRC-associated lipids, amino acids, purine derivatives and other small molecules. We have also identified several microbiota–metabolite associations which may be of mechanistic importance in CRC development and propagation. Surprisingly, in contrast to the association seen between microbiota clusters and prognosis, no such association was found between metabolite clusters and disease-free survival. A possible explanation is that the unbiased hierarchical clustering approach used splits the metabolite data into only two groups; it may be that prognostically important sub-groups of metabolites are not highlighted with this method.

Our study has some key strengths. Samples and clinical data were collected prospectively, and our state-of-the-art bioinformatic approach ensures that the full complexity of the CRC ecological niche is captured while extensively accounting for a multitude of potential confounding factors in the analysis. Cancer driver mutation and exome sequencing data have also been incorporated in the analysis, and clinical follow-up extending to a median duration of 50 months allows for meaningful analysis of patient outcomes following CRC primary resection. We also acknowledge limitations of our study. Although we have validated the results of UK CRC patient microbiota and metabolite clustering analyses in an independently recruited sample set from the Czech Republic, clinical follow-up in the Czech cohort was not sufficient to corroborate the links made between microbiota clusters and disease-free survival in the UK cohort. Owing to our interest in microbiota–host interactions in the tumour microenvironment, we focussed modelling on the CRC mucosal microbiota rather than the faecal stream, and paired normal mucosa (rather than normal mucosa from healthy control patients) was used as the control sample. It has been suggested that repeated rarefication of microbiota sequencing data without replacement may lead to more robust representation of observed sequences [52], and this was not performed in our study. Although our rich metabolomic dataset represents the ultimate functional readout of host–microbiota interactions, we do not have shotgun metagenomic data in this study, which somewhat limits the information we can garner on microbiota functional predictions. Finally, our tumour genetic data are not complete for all samples, and we do not have immune data, which might have allowed further interpretation of our results in the context of host responses.

## Conclusions

In conclusion, our prospective study supports a role for the gut microbiota in clinical outcomes in patients undergoing primary resection for CRC, independent of variables such as AJCC stage, tumour location and adjuvant therapy. We have also identified tumour–microbiota co-metabolites, which warrant further investigation as potential mediators of disease outcome. Future studies should focus on establishing mechanisms through which communities of CRC mucosa-associated commensals, and their metabolic output, might influence determinants of disease-free survival, including immune surveillance.

## Supplementary Information

**Additional file 1:** **Table S1.** Total number of sequencing reads before and after QC filtering and coverage after QC filtering. Median DNA yield per sample: 4.76 µg/ml (interquartile range 2.53 µg/ml – 11.50 µg/ml). **Table S2.** Czech cohort demographics. **Table S3.** Cox proportional hazards analysis showing univariable associations of variables with outcome (death or recurrence of CRC) in 127 UK patients. **Table S4.** Demographics of patients included in full tumour exome sequencing. **Fig. S1.** Czech data set. Y-axis labels are species, or higher taxonomic rank if species data is not known, X-axis labels show the taxonomic order. Clustering from UK data is applied. **Fig. S2.** Scatter plots of matched 16S rRNA amplicon sequencing read counts with bacteria-specific qPCR for four target bacteria (a) Ruminococcus gnavus, (b) Faecalibacterium prausnitzii, (c) Fusobacterium nucleatum, (d) Granulicatella adiacens. r and p values derived from Spearman correlation. **Fig. S3.** Czech data set for metabolites with clustering from UK data applied. **Fig. S4.** Network analysis linking bacterial taxa with metabolites based on identification of metabolites which are involved in enzymatic reactions encoded by genes present in the microbial clusters, with reference to the KEGG database. ^13^ Green circle: the microbe is found in the list of organisms with enzymatic link(s) to the metabolite. Magenta cross: the microbe is not be found in the list of organisms with enzymatic link(s) to the metabolite. **Fig. S5.** Results of full exome sequencing on 9 tumour samples. Each sample is denoted by its dominant microbiota (micro) and metabolomic (met) cluster subtypes. Mutation loads are shown. Mutations to key driver genes are listed within sub-groups. The type of mutation is shown adjacent (right side) of each gene. FS: frameshift deletion; NS: non synonymous mutation; SS: splice site; PS: premature stop; non-FS del: non-frameshift deletion.

## Data Availability

The data generated or analysed during this study are included in this published article, its supplementary information files and in public repositories (https://doi.org/10.5281/zenodo.7326674). The computational workflow for the clustering analysis of the microbiome and metabolomics data sets is available on GitHub (https://github.com/jmp111/CRC). 16S rRNA gene amplicon sequencing data from this study (in fastq-format) are publicly available for download at the European Nucleotide Archive (ENA) database using study accession number PRJEB57635 (http://www.ebi.ac.uk/ena/data/view/ PRJEB57635). Exome sequencing data will be publicly available for download at the European Genome-phenome Archive (EGA) using study accession number EGAD00001008784 upon acceptance for publication.
