# Supplementary material for: Pathobionts in the tumour microbiota predict survival following resection for colorectal cancer
Source: Microbiome. 2023 May 8;11:100. doi: 10.1186/s40168-023-01518-w (PMC10165813; doi:10.1186/s40168-023-01518-w)
Supplement: Supplementary file 2 — Additional file 1: Table S1. Total number of sequencing reads before and after QC filtering and coverage after QC filtering. Median DNA yield per sample: 4.76 µg/ml (interquartile range 2.53 µg/ml – 11.50 µg/ml). Table S2. Czech cohort demographics. Table S3. Cox proportional hazards analysis showing univariable associations of variables with outcome (death or recurrence of CRC) in 127 UK patients. Table S4. Demographics of patients included in full tumour exome sequencing. Fig. S1. Czech data set. Y-axis labels are species, or higher taxonomic rank if species data is not known, X-axis labels show the taxonomic order. Clustering from UK data is applied. Fig. S2. Scatter plots of matched 16S rRNA amplicon sequencing read counts with bacteria-specific qPCR for four target bacteria (a) Ruminococcus gnavus, (b) Faecalibacterium prausnitzii, (c) Fusobacterium nucleatum, (d) Granulicatella adiacens. r and p values derived from Spearman correlation. Fig. S3. Czech data set for metabolites with clustering from UK data applied. Fig. S4. Network analysis linking bacterial taxa with metabolites based on identification of metabolites which are involved in enzymatic reactions encoded by genes present in the microbial clusters, with reference to the KEGG database. 13 Green circle: the microbe is found in the list of organisms with enzymatic link(s) to the metabolite. Magenta cross: the microbe is not be found in the list of organisms with enzymatic link(s) to the metabolite. Fig. S5. Results of full exome sequencing on 9 tumour samples. Each sample is denoted by its dominant microbiota (micro) and metabolomic (met) cluster subtypes. Mutation loads are shown. Mutations to key driver genes are listed within sub-groups. The type of mutation is shown adjacent (right side) of each gene. FS: frameshift deletion; NS: non synonymous mutation; SS: splice site; PS: premature stop; non-FS del: non-frameshift deletion. [file 40168_2023_1518_MOESM1_ESM.docx]

**Pathobionts in the tumour microbiome predict survival following resection for colorectal cancer**

**Supplementary Information**

**Methods**

Tissue extractions for metabolomics

Aqueous extraction:

A solution of methanol/water (1:1 v/v) solution was made up and pre-chilled on ice. Tissue samples measuring between 5 and 120 mg were transferred to individual bead-beating tubes and allowed to thaw gently on ice. Five to ten zirconium beads (0.5mm diameter) were added to each sample tube. To ensure normalisation by sample mass, the pre-chilled methanol/water solution was added in proportions of 100 µl solvent per 10 mg tissue (if the tissue sample was <10 mg, 100 µl was used to allow sufficient solvent for further analysis). The samples underwent serial bead-beating steps in a Precellys 24 tissue homogenizer at 6500Hz for 40 s, followed by a 15 s interval, and again at 6500 Hz for a further 40 s. The samples were returned to dry ice for 5 min before repeating the bead-beating cycle. Following bead-beating, the samples were centrifuged at 13,000 x g for 20 min at 4°C to obtain supernatant. A 100 µl aliquot of supernatant was transferred to a new 1.5 ml Eppendorf safe-lock tube (additionally, whenever possible, a duplicate aliquot of supernatant was obtained). After supernatant removal, the tubes containing the residual pellet were returned to dry ice pending organic extraction. The tubes containing supernatant were placed in a Speed-Vac and dried out at 45°C for 4 h. The dried aqueous extract was stored at -80 °C pending reconstitution.

Prior to the HILIC based small molecule UPLC-MS analysis (HILIC-UPLC-MS), the dried aqueous extracts were thawed at room temperature and reconstituted in 200 µl H2O:Acetonitrile (ACN) (5:95) solvent mixture. The tubes were vortexed for 10 s and stored for 72 h at 4 °C. Samples were vortexed briefly again before centrifugation for 10 min at 13,000 x g at 4 °C. Supernatant (150 µl) from each sample was obtained and transferred to 96-well plates. The remaining volume was pooled to create a study reference (SR) quality control sample which was added to the empty wells in the plate for use as quality control sample.

The plates were dried under nitrogen gas flow ready for HILIC-UPLC-MS analysis. All plates were reconstituted on the day of analysis with 150µl of H2O:ACN (5:95) per well. The reconstitution solvent was doped with a mixture of stable isotope labelled compounds as described in Izzi-Engbeaya et al.^1^ Plates were heat-sealed, shaken for two minutes at room temperature and briefly centrifuged before analysis.

Organic extraction:

A solution of methyltertbutylether/methanol (3:1, v/v) was made and pre-chilled on ice. The pre-chilled methyltertbutylether/methanol solution was added to the residual tissue pellet (including zirconium beads), which was saved following aqueous extraction, in proportions of 100 µl solvent per 10 mg tissue (if the tissue sample was <10 mg, 100 µl was used to allow sufficient solvent for further analysis). The samples underwent serial bead-beating steps at 6500 Hz for 40 s, followed by a 15 s interval, and again at 6500 Hz for a further 40 s. The samples were returned to dry ice for 5 min before repeating the bead-beating cycles. Following bead-beating, the samples were centrifuged at 13,000 x g for 20 min at 4 °C to obtain supernatant. A 100 µl aliquot of supernatant was transferred to glass vials and these were dried under nitrogen gas flow. The dried samples were stored at -80°C pending reconstitution.

Prior to the lipid analysis using a reverse phase based UPLC-MS run (lipid profiling), the dried organic extracts were thawed at room temperature and reconstituted in 500 µl H2O/ACN/isopropanol (1:1:3, v/v/v) solvent mixture containing a lipid standard mix.^1^ The samples were vortexed briefly and stored for 72 h at 4°C. Following a further brief vortex, gentle centrifugation was performed at 200 x g for 2 min at 4°C. Supernatant (450 µl) was obtained from each sample and transferred to 96-well 500µl analytical plates. An aliquot (50 µl) was taken from every sample and combined as a SR sample for QC. Analytical plates were dried down under nitrogen gas flow due to rapid evaporation of reconstitution medium during formatting.

For analysis, each well in the plates was reconstituted with 450µl H2O/ACN/isopropanol (1:1:3, v/v/v) solvent mixture, heat-sealed, shaken and centrifuged. An aliquot (100 µl) from each well was transferred into a new 350µl plate for the lipid positive and lipid negative analyses. Seventy-five microlitres of SR was added to the empty wells in the plate for use as quality control sample. Plates were heat-sealed immediately. Lipid positive plates were run immediately after preparation. Lipid negative plates were stored in a -40 °C freezer, pending analysis.

Target bacterial species culture and bacterium-specific qPCR

Bacterium-specific qPCR was performed on 44 tumour samples to quantify four target bacteria: *Ruminococcus gnavus (Rg), Faecalibacterium prausnitzii (Fp), Fusobacterium nucleatum (Fn)* and *Granulicatella adiacens (Ga)*. *Rg, Fn* and *Ga* were cultured using Fastidious Anaerobe Agar (FAA) supplemented with 5% defibrinated horse blood and Fastidious Anaerobe Broth (FAB). *Fp* was cultured using supplemented Brain Heart Infusion (sBHI) agar and broth. BHI was supplemented with yeast extract (5 g/l), D-(+)-maltose (1 g/l), D-(+)-cellobiose (1 g/l) and L-cysteine hydrochloride monohydrate (0.5 g/l).

Target species were cultured from pure stocks onto agar plates and incubated for 48 hours in an anaerobic chamber (10% CO_2_, 10% H_2_, 80% N_2_) set at 37°C and 70% humidity. Cultures were used to inoculate 10 ml of degassed broth and incubated overnight. 1 ml aliquots of the culture were removed for DNA extraction and to calculate the number of Colony Forming Units (CFU) using a modified version of the Miles and Misra method. Briefly, 1 mL of culture was centrifuged to pellet the bacteria and the pellet washed using PBS. The culture was serially diluted and plated onto appropriate agar plates and incubated in an anaerobic chamber for 48 hours. The resultant colonies were counted and the CFU was calculated. DNA was extracted using the E.Z.N.A.® Bacterial DNA Kit (omega) as per manufactures instructions with the following modifications, the HiBind® DNA Mini Columns were primed by washing with 100 µl 3M NaOH and then with 100 µl of dH_2_O. The resulting DNA of known CFU was used as a standard curve for each bacterial species.

A Master Mix containing the following constituents was made up per samples: 8.5 µL Molecular biology grade water, 12.5 µL 2x SYBR Green PCR master mix (final concentration = 1x in PCR mixture) (Applied biosystems), 1 µL forward primer (10 pmol/µl), 1 µL of reverse primer (10 pmol/µl). Primer sequences for the four bacteria were: Rg forward primer: GGACTGCATTTGGAACTGTCAG, reverse primer: AACGTCAGTCATCGTCCAGAAAG^2^; Fp forward primer: GGAGGAAGAAGGTCTTCGG, reverse primer: AATTCCGCCTACCTCTGCACT^3^; Fn forward primer: GGATTTATTGGGCGTAAAGC, reverse primer: GGCATTCCTACAAATATCTACGAA^4^; Ga forward primer: CAAGCTTCTGCTGATGGATGGA, reverse primer: CTCAGGTCGGCTATGCATCAC^5^.

All samples were run in triplicate using 2 µL of DNA against standard curves for the four target bacteria (from 30000000 to 30 copies of 16S DNA). qPCR cycle conditions were as follows:

- 95ºC for 10 minutes
  - 40 cycles of the following:
- 95ºC for 15 seconds
- 60ºC (Fn) / 64°C (Ga, Fp, Rg) for 60 seconds
  - Hold at 4ºC

Spearman correlations were performed of qPCR 16S rRNA gene copy numbers versus 16S rRNA gene read counts for the four OTUs assigned as Fn, Ga, Fp and Rg respectively. Scatter plots with r and p values are shown in Figure S3 a-d.

Tumour Exome sequencing

Tumour and matched germline DNA were sequenced by the NGS-sequencing facility at the Institute of Cancer Research (ICR). Exome sequencing libraries were prepared using the Agilent SureSelectXT Human All Exon kit according to the manufacturer’s protocol. Paired-end sequencing was performed on an Illumina HiSeq 2500 with a target depth of 100X. Sequences were aligned to the hg19 reference genome using BWA-MEM^6^ (v0.7.12) and duplicates were removed using Picard Tools (<http://picard.sourceforge.net>) (v2.1.0) *MarkDuplicates*. MuTect^7^ (v1.1.7) and VarScan2^8^ (v2.4.1) were used to call somatic single nucleotide variants (SNV) and these were pooled. Platypus^9^ (v0.8.1) was used to identify Indel calls. A similar somatic mutation analysis pipeline has been described previously^10^ and the following study specific steps were used here.

Varscan2: SAMtools (v1.3) *mpileup* was run with minimum mapping quality 1 and minimum base quality 20 and the output directed to VarScan2 *somatic*. Calls were then filtered for a minimum variant allele frequency (VAF) of 5% and converted to BED file format. BAM-readcount (<https://github.com/genome/bam-readcount>) (v0.7.4) was run on the resulting loci with minimum mapping quality 1. The readcounts were then used as input for further filtering using the *fpfilter*.*pl* accessory script.^11^

Mutect: Somatic SNVs were independently identified using MuTect run on default settings. Calls were post-filtered using a minimum 5% VAF.

Platypus: Indel calls were identified using Platypus *callVariants* run on default settings. Any calls with the following flags were excluded - ‘GOF, ‘badReads, ‘hp10,’ MQ’, ‘strandBias’,’ QualDepth’,’ REFCALL’. Somatic indels required the normal genotype to be “0/0”, minimum depth ≥10 in the germline sample, minimum depth ≥20 in the tumour and ≥5 variant reads in the tumour.

Variant calls from Varscan2, Mutect and Platypus were combined and annotated using annovar^12^ (v20160201). Read counts of all SNVs were recalculated using BAM-readcount with a minimum base quality 5.

**Results**

Table S1: Total number of sequencing reads before and after QC filtering and coverage after QC filtering. Median DNA yield per sample: 4.76 µg/ml (interquartile range 2.53 µg/ml – 11.50 µg/ml)

| **Sample** | **Total number of reads (before QC filtering)** | **Number of reads remaining (after QC filtering)** | **Coverage (%)** |
| --- | --- | --- | --- |
| 11 | 25995 | 19527 | 99.95 |
| 12 | 38350 | 28433 | 99.87 |
| 17 | 18443 | 13283 | 99.96 |
| 18 | 11670 | 10578 | 99.99 |
| 19 | 17037 | 15293 | 99.97 |
| 20 | 30717 | 28171 | 99.92 |
| 24 | 14290 | 12826 | 100.00 |
| 25 | 11647 | 10217 | 99.99 |
| 32 | 20886 | 18340 | 99.97 |
| 33 | 26776 | 20148 | 99.88 |
| 34 | 15303 | 12270 | 99.84 |
| 36 | 13925 | 12214 | 99.97 |
| 37 | 18814 | 15094 | 99.88 |
| 44 | 17021 | 13889 | 99.92 |
| 45 | 13652 | 11787 | 99.96 |
| 58 | 16765 | 14944 | 99.97 |
| 60 | 21315 | 19014 | 99.99 |
| 69 | 21550 | 17495 | 99.91 |
| 74 | 20822 | 16939 | 99.89 |
| 76 | 21377 | 17241 | 99.89 |
| 77 | 22528 | 18514 | 99.91 |
| 79 | 25551 | 20125 | 99.85 |
| 80 | 23010 | 18330 | 99.96 |
| 86 | 20661 | 18231 | 99.89 |
| 87 | 14507 | 12286 | 99.93 |
| 88 | 12160 | 10384 | 99.96 |
| 89 | 18599 | 14240 | 99.91 |
| 90 | 18410 | 14205 | 99.92 |
| 92 | 19609 | 15438 | 99.92 |
| 110 | 22976 | 19345 | 99.97 |
| 111 | 19002 | 14546 | 99.99 |
| 146 | 20679 | 17717 | 99.96 |
| 153 | 18407 | 13895 | 99.92 |
| 154 | 19431 | 14975 | 99.89 |
| 160 | 12354 | 11283 | 100.00 |
| 161 | 13885 | 11579 | 99.93 |
| 175 | 24568 | 20526 | 99.89 |
| 184 | 24334 | 20091 | 99.91 |
| 195 | 27301 | 22906 | 99.93 |
| 196 | 26691 | 23593 | 99.93 |
| 197 | 15098 | 11094 | 99.85 |
| 198 | 18676 | 16570 | 99.88 |
| 207 | 17407 | 12975 | 99.89 |
| 209 | 28392 | 22093 | 99.88 |
| 210 | 24392 | 21051 | 99.80 |
| 212 | 19882 | 16162 | 99.80 |
| 216 | 19734 | 15805 | 99.95 |
| 217 | 13309 | 10571 | 99.91 |
| 219 | 21429 | 18940 | 99.95 |
| 223 | 26518 | 23046 | 99.92 |
| 224 | 14337 | 10915 | 99.96 |
| 229 | 32210 | 24148 | 99.84 |
| 232 | 23541 | 19049 | 99.97 |
| 234 | 25151 | 17201 | 99.84 |
| 235 | 31326 | 26289 | 99.83 |
| 236 | 25454 | 21958 | 99.75 |
| 237 | 22431 | 18315 | 99.92 |
| 238 | 27915 | 22689 | 99.91 |
| 239 | 15518 | 12334 | 99.85 |
| 240 | 24612 | 19431 | 99.72 |
| 247 | 16373 | 12503 | 99.91 |
| 248 | 24169 | 17704 | 99.87 |
| 250 | 23772 | 18324 | 99.93 |
| 252 | 22034 | 19263 | 99.85 |
| 254 | 44527 | 39542 | 99.89 |
| 255 | 13554 | 11021 | 99.89 |
| 256 | 34883 | 28036 | 99.85 |
| 257 | 13644 | 9033 | 99.81 |
| 258 | 21886 | 19732 | 99.92 |
| 260 | 18474 | 15732 | 99.77 |
| 261 | 20297 | 14952 | 99.76 |
| 262 | 42094 | 33317 | 99.84 |
| 269 | 20694 | 16501 | 99.91 |
| 270 | 18799 | 13414 | 99.84 |
| 274 | 23509 | 19419 | 99.95 |
| 275 | 17665 | 14993 | 99.89 |
| 277 | 19717 | 15981 | 99.81 |
| 278 | 21115 | 16880 | 99.89 |
| 280 | 18493 | 13907 | 99.87 |
| 281 | 20492 | 17130 | 99.89 |
| 284 | 24163 | 20090 | 99.88 |
| 286 | 21326 | 18460 | 99.87 |
| 287 | 19471 | 16048 | 99.91 |
| 289 | 10838 | 9164 | 99.96 |
| 296 | 21537 | 18642 | 99.80 |
| 297 | 23472 | 22038 | 99.93 |
| 303 | 20692 | 18402 | 99.96 |
| 306 | 12637 | 11074 | 99.99 |
| 308 | 17611 | 13710 | 99.91 |
| 309 | 23591 | 19513 | 99.87 |
| 311 | 18451 | 15054 | 99.95 |
| 312 | 18799 | 15261 | 99.83 |
| 317 | 64738 | 55939 | 99.91 |
| 318 | 28856 | 24866 | 99.92 |
| 321 | 13324 | 10134 | 99.85 |
| 322 | 19196 | 15068 | 99.85 |
| 357 | 23088 | 18976 | 99.89 |
| 358 | 32955 | 25479 | 99.77 |
| 375 | 13656 | 11516 | 99.87 |
| 376 | 13183 | 11914 | 99.93 |
| 378 | 18347 | 11360 | 99.87 |
| 379 | 39562 | 28758 | 99.71 |
| 384 | 11524 | 10131 | 99.93 |
| 386 | 13067 | 11754 | 99.97 |
| 387 | 37133 | 29816 | 99.92 |
| 388 | 20834 | 13684 | 99.93 |
| 390 | 18806 | 13177 | 99.84 |
| 392 | 13140 | 9344 | 99.84 |
| 394 | 15554 | 10052 | 99.85 |
| 395 | 23623 | 19268 | 99.85 |
| 396 | 20697 | 18159 | 99.96 |
| 415 | 16362 | 11591 | 99.84 |
| 416 | 24563 | 18456 | 99.92 |
| 417 | 27754 | 15096 | 99.96 |
| 419 | 20331 | 15364 | 99.85 |
| 420 | 31194 | 30096 | 99.99 |
| 423 | 14177 | 11667 | 99.95 |
| 424 | 23646 | 20912 | 99.91 |
| 425 | 23376 | 19491 | 99.96 |
| 426 | 31743 | 24519 | 99.83 |
| 427 | 26610 | 20555 | 99.84 |
| 428 | 20684 | 16583 | 99.95 |
| 429 | 18737 | 14508 | 99.92 |
| 430 | 42704 | 15813 | 99.91 |
| 431 | 9544 | 7614 | 99.93 |
| 432 | 23760 | 12843 | 99.93 |
| 433 | 19816 | 18028 | 99.92 |
| 434 | 21012 | 16924 | 99.91 |
| 435 | 20143 | 15116 | 99.65 |
| 436 | 22220 | 16718 | 99.89 |
| 437 | 9095 | 7620 | 99.95 |
| 438 | 40219 | 34000 | 99.84 |
| 439 | 16410 | 13308 | 99.88 |
| 441 | 22144 | 17493 | 99.93 |
| 442 | 26268 | 21745 | 99.91 |
| 443 | 22600 | 19508 | 99.89 |
| 445 | 20008 | 15590 | 99.97 |
| 446 | 26153 | 24975 | 99.95 |
| 447 | 12312 | 8834 | 99.80 |
| 448 | 24352 | 16358 | 99.81 |
| 464 | 22034 | 17474 | 99.89 |
| 465 | 16111 | 13179 | 99.80 |
| 468 | 26147 | 21585 | 99.92 |
| 469 | 23402 | 18474 | 99.92 |
| 470 | 23117 | 19147 | 99.91 |
| 471 | 22412 | 18510 | 99.85 |
| 472 | 22169 | 16650 | 99.81 |
| 473 | 12334 | 8343 | 99.85 |
| 474 | 20509 | 16394 | 99.85 |
| 493 | 12487 | 10655 | 100.00 |
| 494 | 10903 | 9579 | 99.99 |
| 530 | 16212 | 12619 | 99.87 |
| 531 | 18689 | 16434 | 99.88 |
| 532 | 15878 | 12707 | 99.85 |
| 533 | 16192 | 13695 | 99.97 |
| 534 | 35728 | 15002 | 99.81 |
| 535 | 18883 | 7636 | 99.92 |
| 536 | 16110 | 13476 | 99.92 |
| 537 | 15721 | 12717 | 99.91 |
| 538 | 21076 | 17781 | 99.88 |
| 539 | 19992 | 16384 | 99.84 |
| 540 | 19781 | 15183 | 99.99 |
| 541 | 25497 | 20100 | 99.76 |
| 542 | 17023 | 13256 | 99.96 |
| 543 | 23873 | 19698 | 99.96 |
| 544 | 16746 | 14001 | 99.93 |
| 545 | 26915 | 21530 | 99.87 |
| 546 | 13348 | 11459 | 99.96 |
| 548 | 32588 | 29906 | 99.89 |
| 550 | 29832 | 24762 | 99.92 |
| 552 | 27125 | 26034 | 99.95 |
| 553 | 25486 | 23033 | 99.95 |
| 554 | 33046 | 29792 | 99.85 |
| 555 | 18787 | 13451 | 99.88 |
| 556 | 21810 | 19786 | 100.00 |
| 557 | 34647 | 29449 | 99.84 |
| 558 | 26948 | 17055 | 99.92 |
| 560 | 10114 | 9243 | 99.96 |
| 561 | 29297 | 22895 | 99.69 |
| 563 | 33576 | 17097 | 99.87 |
| 564 | 26738 | 23416 | 99.95 |
| 565 | 52238 | 43211 | 99.92 |
| 566 | 14452 | 10995 | 99.95 |
| 567 | 10883 | 9687 | 99.89 |
| 568 | 24043 | 16359 | 99.76 |
| 569 | 38680 | 30293 | 99.88 |
| 571 | 17268 | 13828 | 99.96 |
| 572 | 24285 | 18952 | 99.81 |
| 573 | 28460 | 23922 | 99.79 |
| 576 | 17528 | 15090 | 99.99 |
| 577 | 12652 | 10988 | 99.96 |
| 578 | 11226 | 9453 | 99.96 |
| 580 | 28631 | 25033 | 99.93 |
| 581 | 35904 | 31308 | 99.91 |
| 582 | 26508 | 21995 | 99.95 |
| 583 | 29688 | 25183 | 99.89 |
| 589 | 11350 | 9939 | 99.97 |
| 590 | 16492 | 14304 | 99.89 |
| 591 | 38331 | 29970 | 99.87 |
| 592 | 24125 | 20776 | 99.93 |
| 593 | 16437 | 14486 | 99.89 |
| 594 | 18207 | 15157 | 99.89 |
| 607 | 81654 | 64113 | 99.89 |
| 608 | 32700 | 27798 | 99.97 |
| 609 | 22046 | 18903 | 99.96 |
| 610 | 10400 | 9270 | 99.96 |
| 611 | 31938 | 29582 | 99.99 |
| 612 | 26083 | 24041 | 99.93 |
| 619 | 15992 | 14136 | 99.89 |
| 620 | 15088 | 11024 | 99.91 |
| 631 | 50250 | 42960 | 99.95 |
| 632 | 94344 | 79920 | 99.95 |
| 633 | 38181 | 30758 | 99.95 |
| 634 | 83003 | 70545 | 99.92 |
| 635 | 51698 | 42671 | 99.84 |
| 636 | 65795 | 57241 | 99.85 |
| 638 | 52140 | 43182 | 99.93 |
| 639 | 38433 | 23775 | 99.92 |
| 642 | 46383 | 36017 | 99.87 |
| 643 | 49620 | 36899 | 99.93 |
| 650 | 39835 | 31413 | 99.95 |
| 651 | 40954 | 33321 | 99.91 |
| 657 | 27270 | 17568 | 99.92 |
| 658 | 61771 | 53575 | 99.89 |
| 661 | 48511 | 28933 | 99.89 |
| 662 | 64875 | 52645 | 99.92 |
| 663 | 32872 | 25184 | 99.91 |
| 664 | 46693 | 32205 | 99.96 |
| 666 | 32910 | 27509 | 99.99 |
| 668 | 10820 | 7946 | 99.93 |
| 669 | 37610 | 28369 | 99.87 |
| 671 | 22596 | 16430 | 99.93 |
| 672 | 16671 | 14253 | 99.92 |
| 701 | 79502 | 57202 | 99.77 |
| 702 | 81237 | 63194 | 99.77 |
| 706 | 58484 | 42349 | 99.80 |
| 707 | 83039 | 62167 | 99.95 |
| 708 | 74092 | 58966 | 99.91 |
| 709 | 69758 | 51367 | 99.80 |
| 712 | 76239 | 54522 | 99.85 |
| 713 | 27627 | 19286 | 99.88 |
| 714 | 76532 | 61644 | 99.91 |
| 715 | 77265 | 70122 | 99.88 |
| 716 | 53579 | 46669 | 99.84 |
| 717 | 31286 | 23630 | 99.83 |
| 718 | 63937 | 55454 | 99.87 |
| 719 | 58930 | 47716 | 99.80 |
| 720 | 61911 | 39326 | 99.84 |

Table S2: Czech cohort demographics

| N | 62 |
| --- | --- |
| Male:Female | 44:18 |
| Median Age (range) | 68 (46-86) |
| Median BMI | 28.1 |
| Neo-adjuvant Treatment  *None* | 62 |
| Adjuvant chemotherapy | 29 (46.8%) |
| Tumour Site  *Rectum*  *Sigmoid & Recto-sigmoid*  *Descending*  *Splenic Flexure*  *Transverse*  *Hepatic Flexure*  *Caecum & Ascending colon* | 18  14  3  2  4  0  21 |
| T stage  *T1/2*  *T3*  *T4* | 4  49  9 |
| N stage  *N0*  *N1*  *N2* | 39  17  6 |
| M stage  *M0*  *M1* | 56  6 |
| AJCC stage  *I*  *II*  *III*  *IV* | 0  37  19  6 |
| Differentiation  *Well*  *Moderate*  *Poor* | 6  46  8 |

Table S3: Cox proportional hazards analysis showing univariable associations of variables with outcome (death or recurrence of CRC) in 127 UK patients.

| **Variable** | **Hazard ratio** | **95% CI** | **P value** |
| --- | --- | --- | --- |
| Microbiota cluster 1 | 1.25 | 1.1-1.4 | <0.0001 |
| Microbiota cluster 7 | 0.62 | 0.4-0.9 | 0.040 |
| Age | 1 | 0.9-1.0 | 0.71 |
| BMI | 0.99 | 0.9-1.1 | 0.72 |
| Sex | 1 | 0.5-2.0 | 0.92 |
| Use of adjuvant chemotherapy | 0.79 | 0.4-1.6 | 0.54 |
| Anatomical location of tumour | 0.81 | 0.6-1.2 | 0.25 |
| AJCC stage | 2 | 1.2-3.1 | 0.005 |
| EMVI | 1.8 | 0.9-3.5 | 0.07 |
| Tumour differentiation | 0.47 | 0.2-0.9 | 0.04 |
| DNA mismatch repair deficiency^$^ | 2.5 | 1.0-6.2 | 0.04 |

^$^ DNA mismatch repair deficiency classification performed in 79 cases.

Table S4: Demographics of patients included in full tumour exome sequencing

No. Age Gender Ethnicity BMI Tumour location Stage Differentiation

1 74 Male White 32 Ascending II Moderate

2^$^ 71 Male Asian NA Caecum III Moderate

3 83 Male Black 26 Transverse II Moderate

4 85 Male Asian 21 Ascending II Moderate

5 87 Male White 29 Caecum III Moderate

6 85 Female White 31 Caecum II Mod.-Poor

7 70 Male White 39 Ascending I Moderate

8 90 Female White 28 Ascending I Moderate

9 79 Male White 29 Transverse II Moderate

10 67 Female White 17 Transverse II Moderate

^$^sample ultimately excluded from tumour exome sequencing analysis as the sample failed to generate adequate sequencing library.

**Supplementary Figures**


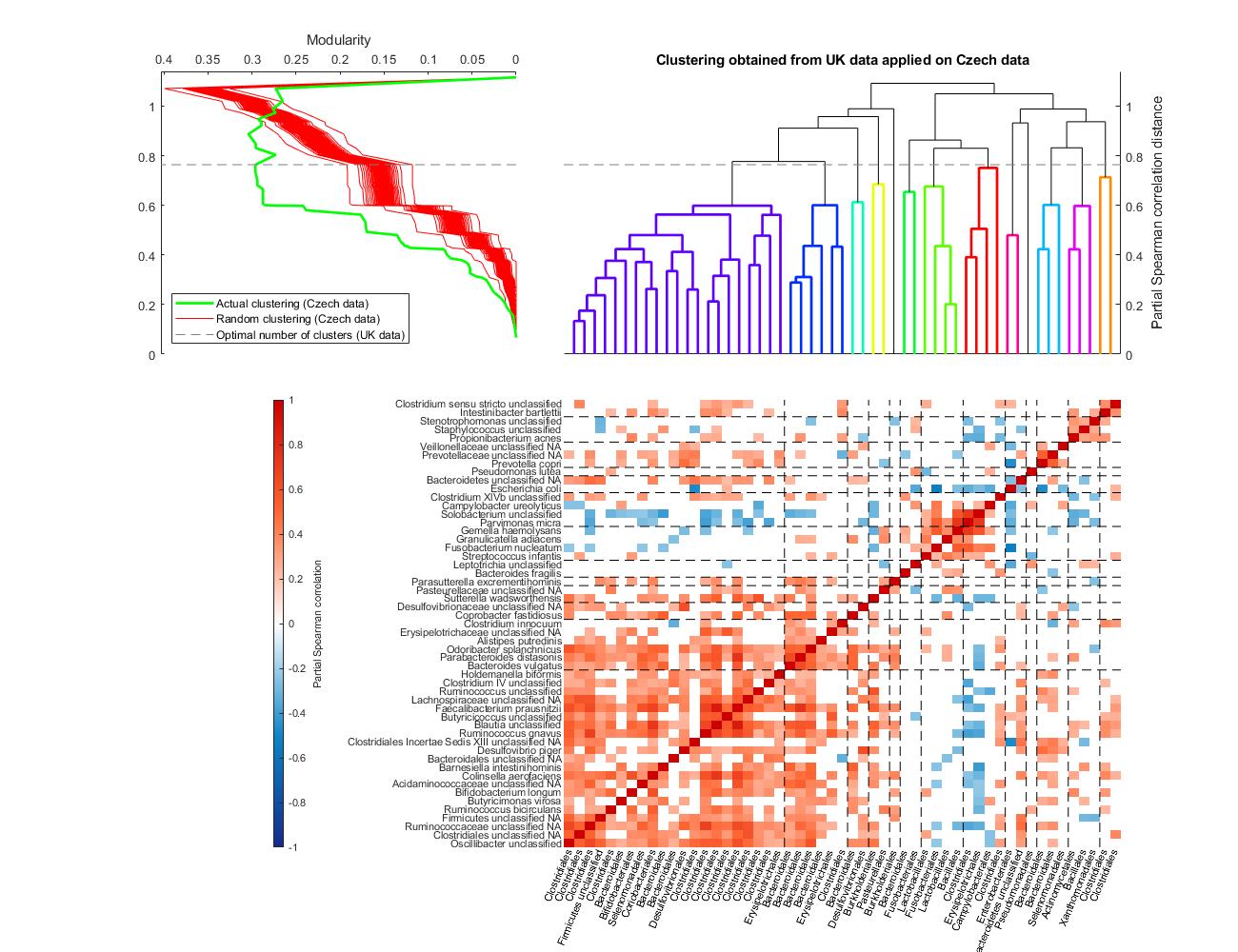


**Figure S1.** Czech data set. Y-axis labels are species, or higher taxonomic rank if species data is not known, X-axis labels show the taxonomic order. Clustering from UK data is applied.

**Figure S2:** Scatter plots of matched 16S rRNA amplicon sequencing read counts with bacteria-specific qPCR for four target bacteria (a) *Ruminococcus gnavus*, (b) *Faecalibacterium prausnitzii*, (c) *Fusobacterium nucleatum,* (d) *Granulicatella adiacens*. r and p values derived from Spearman correlation.


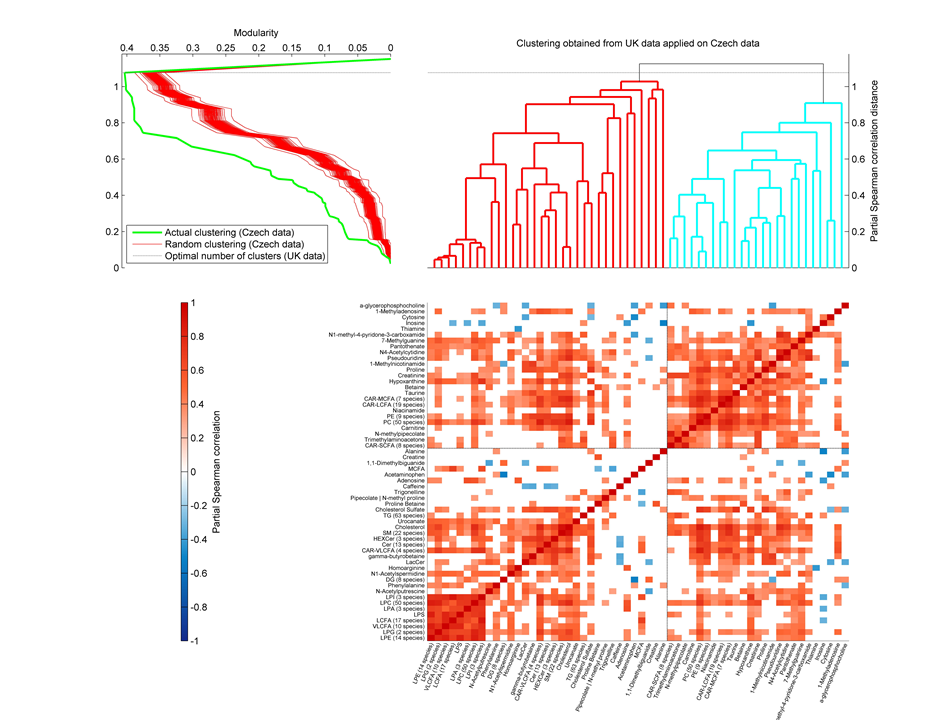


**Figure S3.** Czech data set for metabolites with clustering from UK data applied.


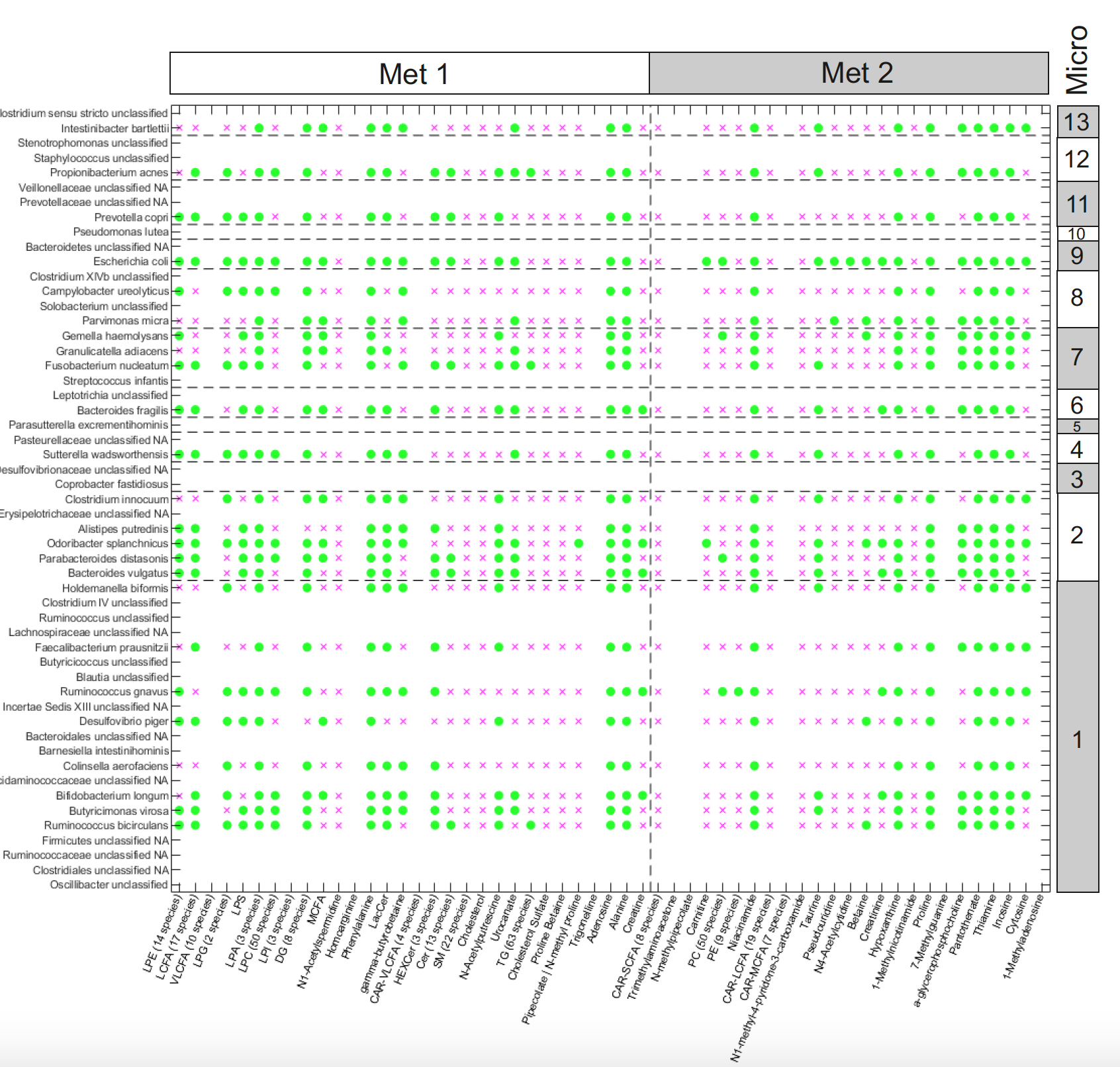


**Figure S4.** Network analysis linking bacterial taxa with metabolites based on identification of metabolites which are involved in enzymatic reactions encoded by genes present in the microbial clusters, with reference to the KEGG database.^13^ Green circle: the microbe is found in the list of organisms with enzymatic link(s) to the metabolite. Magenta cross: the microbe is not be found in the list of organisms with enzymatic link(s) to the metabolite.

**Figure S5:** Results of full exome sequencing on 9 tumour samples. Each sample is denoted by its dominant microbiota (micro) and metabolomic (met) cluster subtypes. Mutation loads are shown. Mutations to key driver genes are listed within sub-groups. The type of mutation is shown adjacent (right side) of each gene. FS: frameshift deletion; NS: non synonymous mutation; SS: splice site; PS: premature stop; non-FS del: non-frameshift deletion.

References

1. Izzi-Engbeaya C, Comninos AN, Clarke SA, et al. The effects of kisspeptin on beta-cell function, serum metabolites and appetite in humans. Diabetes Obes Metab 2018;20:2800-2810.

2. Png CW, Linden SK, Gilshenan KS, et al. Mucolytic bacteria with increased prevalence in IBD mucosa augment in vitro utilization of mucin by other bacteria. Am J Gastroenterol 2010;105:2420-8.

3. Hedin CR, McCarthy NE, Louis P, et al. Altered intestinal microbiota and blood T cell phenotype are shared by patients with Crohn's disease and their unaffected siblings. Gut 2014;63:1578-86.

4. Kinross J, Mirnezami R, Alexander J, et al. A prospective analysis of mucosal microbiome-metabonome interactions in colorectal cancer using a combined MAS 1HNMR and metataxonomic strategy. Sci Rep 2017;7:8979.

5. Farrell JJ, Zhang L, Zhou H, et al. Variations of oral microbiota are associated with pancreatic diseases including pancreatic cancer. Gut 2012;61:582-8.

6. Li H, Durbin R. Fast and accurate short read alignment with Burrows-Wheeler transform. Bioinformatics 2009;25:1754-60.

7. Cibulskis K, Lawrence MS, Carter SL, et al. Sensitive detection of somatic point mutations in impure and heterogeneous cancer samples. Nat Biotechnol 2013;31:213-9.

8. Koboldt DC, Zhang Q, Larson DE, et al. VarScan 2: somatic mutation and copy number alteration discovery in cancer by exome sequencing. Genome Res 2012;22:568-76.

9. Rimmer A, Phan H, Mathieson I, et al. Integrating mapping-, assembly- and haplotype-based approaches for calling variants in clinical sequencing applications. Nature Genetics 2014;46:912.

10. Woolston A, Khan K, Spain G, et al. Genomic and Transcriptomic Determinants of Therapy Resistance and Immune Landscape Evolution during Anti-EGFR Treatment in Colorectal Cancer. Cancer Cell 2019;36:35-50 e9.

11. Koboldt DC, Larson DE, Wilson RK. Using VarScan 2 for Germline Variant Calling and Somatic Mutation Detection. Curr Protoc Bioinformatics 2013;44:15.4.1-17.

12. Wang K, Li M, Hakonarson H. ANNOVAR: functional annotation of genetic variants from high-throughput sequencing data. Nucleic Acids Research 2010;38:e164-e164.

13. Kanehisa M, Goto S. KEGG: kyoto encyclopedia of genes and genomes. Nucleic Acids Res 2000;28:27-30.
